# Setting sail for Paris 2024: Retrospective analysis of world‐class ILCA 7 Olympic sailors’ cardiorespiratory fitness (2015–2020)

**DOI:** 10.1113/EP091913

**Published:** 2024-07-18

**Authors:** Damir Zubac, Zoran Valić, Vladimir Ivančev

**Affiliations:** ^1^ Department 1 of Internal Medicine Center for Integrated Oncology Aachen University Hospital of Cologne Cologne Germany; ^2^ Science and Research Center Koper Institute for Kinesiology Research Koper Slovenia; ^3^ Department of Integrative Physiology, School of Medicine University of Split Split Croatia; ^4^ Faculty of Kinesiology University of Split Split Croatia

**Keywords:** Olympics, oxygen uptake, sailing

## Abstract

The aim of this retrospective analysis was to provide a more comprehensive understanding of the cardiorespiratory profile of world‐class ILCA‐7 sailors (*n* = 3, all males), through a longitudinal evaluation offering real‐world data on physiological profile and exercise intensity domains. The cardiopulmonary exercise testing (CPET) was performed by the same researchers using the same equipment during the study. Assessments took place twice a year, aligning with major international competition preparations. Participants trained and competed at the same sailing club in Split, Croatia, under consistent supervision from the same team throughout the study, winning a total of 21 medals at major international competitions. The recorded ${\dot{V}}_{O_{2}\mathrm{peak}}$ ranged from 51.7 ± 1.6 to 61.9 ± 3.0 mL min^−1^ kg^−1^, respectively. Similarly, peak power output varied from 352 ± 10 to 426 ± 34 W. The changes in physiological responses at the ventilatory thresholds were proportional to the changes in peak cardiorespiratory fitness capacity. Interestingly, the oxygen pulse measured in 2015 was 25 ± 1 mL O_2_ beat^−1^. Over the subsequent 6 years, the O_2_ pulse marginally increased and appeared to stabilize at 27 ± 1 mL O_2_ beat^−1^ in 2020, when these athletes were 32 ± 3 years old. This work offers a broader understanding of world‐class Olympic sailors’ cardiorespiratory fitness, going beyond the standard assessment of peak ${\dot{V}}_{O_{2}}$ to incorporate an analysis of ventilatory thresholds. While a direct link between cardiorespiratory fitness and competitive success remains ambiguous, the importance of a well‐rounded aerobic capacity for excellence in ILCA‐7 sailing class is evident.

## Abstract (highlights)

- **What is the central question of this study?**
  What are the temporal changes in the physiological profiles of three world‐class ILCA‐7 sailors?
- **What is the main finding and its importance?**
  Data on oxygen pulse adjustments suggest the involvement of compensatory cardiovascular mechanisms, likely associated with the isometric and quasi‐isometric contractions inherent in ILCA‐7 sailing. This is evidenced by the absence of an age‐related increase in oxygen pulse, a phenomenon often observed in endurance athletes throughout their competitive careers.

## 1 INTRODUCTION

The ILCA‐7, a monohulled sailing category manned by a single athlete, made its inaugural appearance at the 1996 Atlanta Summer Olympics. Since then, it has been a consistent presence in subsequent Olympic Games, establishing itself as a longstanding and prominent event in Olympic sailing. ILCA‐7 sailors, known for their agile hiking skills, expertly manoeuvre the boat in dynamic conditions, thanks to their adept management of the sailor‐to‐yacht weight ratio and the frequent challenges of weather conditions (Bojsen‐Møller et al., 2015; Winchcombe et al., 2021). The Olympic sailing regattas for the ILCA‐7 typically span 5–7 days, with two to three races conducted daily, contingent upon weather conditions, and sailors generally participate in 10–12 races throughout the series, including the medal race. The rationale behind this structure is to subject sailors to a comprehensive test of skills across diverse wind and sea conditions, mitigating the impact of individual race unpredictability and ensuring that overall results reflect consistent performance. Analysis of Global Positioning Satellite (GPS) data from the 2019–2020 Hempel World Cup Series and the 2020 Tokyo Olympic Games revealed that high‐level ILCA‐7 races have an approximate duration of 50 min, covering distances between 5.5 and 9.5 km per race (Pan & Sun, 2022). The relatively brief duration of each race necessitates high‐level physical exertion, particularly influenced by challenging weather conditions (Caraballo et al., 2019; Casadio et al., 2016; García & Martínez, 2015). Indeed, ILCA‐7 sailors need to control the balance of the boat in continuously varying wind and wave conditions (Bourgois et al., 2017), especially during the ‘hiking’ position, which accounts for 60%–93% of the total upwind time. This position, involving a quasi‐isometric bilateral and multi‐joint movement during a race, exerts substantial physiological stress on the sailor. Moreover, hiking performance is strongly related to the development of neuromuscular fatigue in the quadriceps femoris and plays a pivotal role in overall sailing performance (Bourgois et al., 2017). Despite the global interest in Olympic sailing, studies on the cardiometabolic profile of sailors remain limited, due to challenges in conducting valid on‐water data recording. While measurements of physiological responses to laboratory‐based sailing simulations and emulations are reported in the literature (Bojsen‐Møller et al., 2015; Cunningham & Hale, 2007; Bourgois et al., 2016; Callewaert et al., 2013), proxy data based on actual physiological demands during sail racing are sparse. Interestingly, data on cardiovascular demands during on‐water sailing are rarely reported, with only Lopez et al. (2016) suggesting almost two‐fold greater mean arterial pressure (MAP) responses (and subsequently total peripheral resistance) during actual sailing compared to laboratory testing. This elevated cardiovascular response is likely attributable to the significant physiological demands imposed by the hiking position of the sailor. However, due to the cross‐sectional nature of Lopez's study, comprehensive data regarding cardiovascular adaptations are lacking. Early studies by Legg et al. (1997) involving sailors in the 1996 Olympic Games revealed inconsistent correlations between aerobic fitness and racing results in both light and heavy wind regattas. While aerobic training and fitness have demonstrated a direct link to sailors’ reaction speed to wind shifts, endurance, decision‐making and concentration (Allen & de Jong, 2006), especially in later race stages, individual identification of optimal metabolic zones and an accurate prescription of equivalent targeted constant work‐rate exercise from lab‐based cardiopulmonary exercise testing (CPET) are still lacking. Most prior research on world‐class sailors primarily presented data from single‐visit CPET, without details on the rate of oxygen uptake (${\dot{V}}_{O_{2}}$) at ventilatory thresholds. Furthermore, information regarding cardiorespiratory adaptations during training and detraining periods is limited (Ishida et al., 2023), and consequently, the extent of adaptation in ${\dot{V}}_{O_{2}}$ or any other physiological indicator of cardiometabolic demands associated with sailing remains relatively unknown. This is particularly noteworthy, especially considering the necessity for offering personalized training interventions to optimize both cardiorespiratory fitness (Gifford & Larsen, 2023) and overall performance. This study aims to provide a more comprehensive insight into the cardiorespiratory profile of a unique population—world‐class ILCA‐7 sailors who had previously won medals at major international competition.

## 2 METHODS

### 2.1 Participants

All athletes provided written consent to participate in this study. The data collection protocols received approval from the Institutional Research Ethics Board and were conducted in compliance with the principles outlined in the latest version of the *Declaration of Helsinki*. This longitudinal observation reports the results of three world‐class ILCA‐7 sailors (age 26 ± 3, all males, body mass 81.6 ± 0.5 kg, body height 180.7 ± 2.5 cm at the initial laboratory visit) in the period from 2015 to 2020. Throughout their competitive careers, these athletes have won 40 medals at major international competitions, including the Olympic Games, World Championships, European Championships and Mediterranean Games, with 21 of these medals collected between 2015 and 2020.

### 2.2 Study design and data collection protocols

All data were collected at the Exercise Physiology Laboratory, Faculty of Kinesiology, University of Split, by the same researchers using the same equipment throughout the research period. All athletes trained and competed from the same sailing club in Split, Croatia, under the supervision of the same sailing coach, physical fitness coach, nutritionist, physiotherapist and medical doctor. The assessments occurred semi‐annually, typically in June (a) and November (b). However, for the baseline testing in June 2015, data were collected only once due to a dense competition calendar that necessitated trans‐continental journeys. All data collection protocols were executed in the morning hours, between 08:30 and 10:30, under standardized laboratory conditions. More precisely, preceding each CPET, anthropometric characteristics, resting ECG, blood pressure and spirometry were recorded, in accordance with established guidelines (Drezner, 2012; Miller et al., 2005). The training regimen encompassed three distinct phases: preparatory, competitive and transition periods. It entailed approximately 380–480 h of on‐water sailing annually, supplemented by 135–150 additional hours of gym work. The training regime involved a blend of moderate and high‐intensity workouts, adjusted according to the competition calendar. During the study period, they underwent regular doping controls, all of which yielded negative results. Reports of injuries and infections, including the Covid‐19 outbreak, were documented by the team physician (V.I.) and physiotherapist.

### 2.3 Cardio‐pulmonary exercise testing

The pulmonary gas exchange was assessed on a breath‐by‐breath basis using a stationary cardiopulmonary exercise testing unit (Quark, Cosmed, Rome, Italy), which was synchronized with the cycle‐ergometer (Ergoline 900, Hamburg, Germany). Calibration of the metabolic chart was performed before each laboratory session, following the manufacturer's guidelines, utilizing a gas mixture of known composition and ambient air, as outlined elsewhere (Zubac et al., 2021). Prior to data collection, their height and weight were measured and thereafter all participants underwent a period of quiet rest on the bed, during which they were equipped with a silicone mask (Hans Rudolph, Shawnee, KS, USA) connected to a metabolic analyser, and an HR belt was securely positioned around the chest (Garmin device, HRM‐3 SS, Olathe, KS, USA). All CPET protocols were supervised by the same research staff, using the same equipment throughout data collection. In brief, following a 5‐min warm‐up at 50 W, each participant underwent an incremental ramp test (25 W min^−1^) on a cycle‐ergometer until reaching task failure. The aim was to determine ${\dot{V}}_{O_{2}\mathrm{peak}}$, peak power output (PO) and maximal heart rate (HR_max_). Task failure was established when the cadence dropped below 75 rpm for more than 10 consecutive seconds, even with verbal encouragement from the research staff.

### 2.4 Data processing and analysis

Determination of exercise intensity domains was as follows. Gas exchange threshold (GET) and respiratory compensation point (RCP) were determined separately by two researches in accordance with standardized, previously published criteria for athletes (Pallarés et al., 2016), and in agreement with the originally proposed V‐slope method (Beaver et al., 1986). Briefly, the GET was visually inspected and established as a point at which a systematic increase in the ventilatory equivalent of the rate of oxygen uptake (${\dot{V}}_{E}$/${\dot{V}}_{O_{2}}$) and end‐tidal pressure of oxygen were observed, without a concomitant increase in the ventilatory equivalent of carbon dioxide production (${\dot{V}}_{E}$/${\dot{V}}_{CO_{2}}$). The RCP was identified based on the criteria of observing simultaneous increases in both ${\dot{V}}_{E}$/${\dot{V}}_{O_{2}}$ and ${\dot{V}}_{E}$/${\dot{V}}_{CO_{2}}$, along with a systematic decrease in end‐tidal carbon dioxide pressure ($P_{\mathrm{ETC}O_{2}}$) for corresponding ${\dot{V}}_{O_{2}}$. In addition, the influence of delayed ${\dot{V}}_{O_{2}}$ was corrected for each participant (using the mean response time) to calculate the PO associated with ${\dot{V}}_{O_{2}}$ at GET (Boone & Bourgois, 2012). The ${\dot{V}}_{O_{2}\mathrm{peak}}$ was determined using the moving average (20‐s interval) of data recorded during the last minute of the incremental ramp test, whereas peak PO and HR_max_. were defined as data reached at the end of the test(s). All data are given as means ± SD. The coefficient of variation (CV, %) was calculated, and a Microsoft Excel spreadsheet was used to process and report the data.

## 3 RESULTS

**FIGURE 1:**
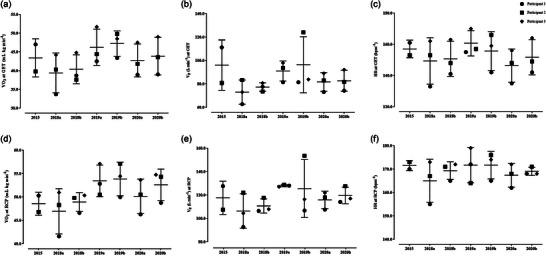
Individual and mean responses to CPET at ventilatory thresholds (*n* = 3).

**FIGURE 2:**
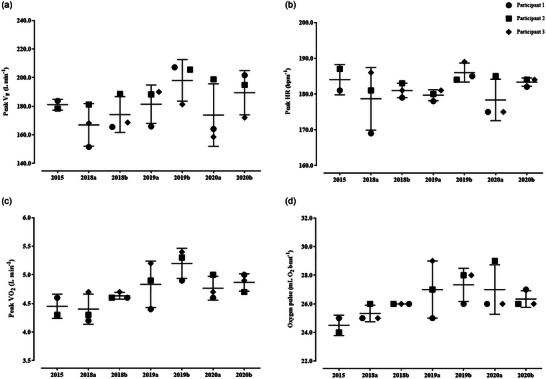
Individual responses to CPET (*n* = 3).

In short, all cardiovascular data collected prior to CPET were within the reference values for athletes. Spirometry parameters, including forced vital capacity (FVC) and maximal voluntary ventilation remained within reference values, with, for example, the FEV_1_/FVC index at 78 ± 6% of predicted values. No major injuries or infections occurred in the period from 2015 to 2020; however, minor incidents of lower back pain and muscle sprains were recorded. Their body mass fluctuated by 1.2% over the course of the study. In Tables 1 and 2, average values of cardiorespiratory fitness data are presented, including peak CPET readings and ventilatory thresholds, while Figures 1 and 2 depict individual data distribution. The CV for peak ${\dot{V}}_{O_{2}}$, PPO and HR_max_ was 3.9%, 9.1% and 2.5% throughout the study, respectively. Slightly higher readings were observed for the same variables at the ventilatory thresholds: GET: 10.8%, 11.6% and 7.1%, respectively; and again for the RCP: 7.6%, 8.9% and 3.6%, respectively. One disadvantage of this work was the fact that the CPET data of one participant from 2015 was lost due to technical problems.

**TABLE 1:** Cardiorespiratory parameters measured at the gas exchange thresholds and respiratory compensation points.

Years	2015	2018a	2018b	2019a	2019b	2020a	2020b
${\dot{V}}_{E}$ (L min^−1^)							
GET	96.0 ± 21.5	73.0 ± 10.3	77.3 ± 3.6	91.0 ± 8.6	96.4 ± 23.9	81.7 ± 8.0	82.7 ± 9.0
RCP	117.7 ± 14.2	106.3 ± 14.7	110.0 ± 6.1	127.9 ± 2.0	125.5 ± 25.0	116.0 ± 7.5	119.5 ± 7.1
${\dot{V}}_{O_{2}}$ (mL kg min^−1^)							
GET	43.4 ± 5.1	39.4 ± 5.3	40.4 ± 3.9	46.2 ± 4.8	47.2 ± 3.4	42.7 ± 4.4	43.9 ± 5.0
RCP	48.6 ± 2.5	46.9 ± 4.8	48.9 ± 2.02	53.4 ± 3.4	53.8 ± 3.6	50.1 ± 3.7	52.6 ± 3.4
% of ${\dot{V}}_{O_{2}\mathrm{peak}}$							
GET	79 ± 7	74 ± 6	72 ± 3	82 ± 4	75 ± 3	72 ± 7	75 ± 9
RCP	87 ± 1	88 ± 5	87 ± 2	90 ± 5	85 ± 3	89 ± 5	89 ± 7
RER							
GET	1.00 ± 0.01	0.91 ± 0.02	0.94 ± 0.02	0.99 ± 0.03	0.95 ± 0.05	0.93 ± 0.03	0.96 ± 0.03
RCP	1.05 ± 0.01	1.03 ± 0.03	1.02 ± 0.02	1.08 ± 0.03	1.03 ± 0.02	1.03 ± 0.01	1.06 ± 0.03
HR (beats min^−1^)							
GET	157 ± 6	148 ± 17	151 ± 11	160 ± 8	155 ± 12	146 ± 11	152 ± 11
RCP	172 ± 2	164 ± 12	169 ± 4	172 ± 8	172 ± 6	167 ± 5	169 ± 2
PO (W kg^−1^)							
GET	3.2 ± 0.4	3.1 ± 0.5	3.2 ± 0.4	3.7 ± 0.4	3.5 ± 0.5	3.3 ± 0.5	3.5 ± 0.4
RCP	3.8 ± 0.1	3.9 ± 0.3	4.0 ± 0.4	4.3 ± 0.4	4.3 ± 0.5	4.1 ± 0.5	4.4 ± 0.3
Phase duration (mm:ss)							
GET	2:30 ± 00:10	2:25 ± 00:07	2:20 ± 1:50	2:30 ± 00:05	3:10 ± 00:10	3:05 ± 00:45	3:30 ± 00:30
RCP	3:35 ± 00:45	2:21 ± 00:17	2:58 ± 00:24	2:25 ± 00:54	3:38 ± 00:41	3:40 ± 00:39	2:57 ± 00:20

*Note*: Number of participants *n* = 3. Abbreviations: GET, gas exchange threshold; HR, heart rate; PO, power output; RCP, respiratory compensation point; RER, respiratory exchange ratio; ${\dot{V}}_{E}$, pulmonary ventilation; ${\dot{V}}_{O_{2}}$, oxygen uptake.

**TABLE 2:** Peak values of cardiorespiratory parameters.

Years	2015	2018a	2018b	2019a	2019b	2020a	2020b
${\dot{V}}_{E}$ (L min^−1^)	181.1 ± 3.8	167.0 ± 14.9	174.1 ± 12.5	181.4 ± 13.5	198.0 ± 14.5	173.8 ± 21.9	189.5 ± 15.5
*f*_R_ peak (breaths min^−1^)	70 ± 9	55 ± 14	55 ± 10	57 ± 12	64 ± 8	57 ± 12	60 ± 8
*V*_T_ peak (L)	3.0 ± 0.1	3.3 ± 0.4	3.3 ± 0.3	3.4 ± 0.4	3.5 ± 0.4	3.4 ± 0.3	3.4 ± 0.4
${\dot{V}}_{O_{2}}$ (L min^−1^)	4.5 ± 0.2	4.4 ± 0.3	4.6 ± 0.1	4.9 ± 0.4	5.2 ± 0.2	4.7 ± 0.3	4.9 ± 0.1
${\dot{V}}_{O_{2}}$ (mL kg min^−1^)	54.7 ± 1.8	51.7 ± 1.6	55.7 ± 0.7	56.8 ± 3.9	61.9 ± 3.05	59.05 ± 4.4	59.2 ± 1.1
O_2_ pulse (mL O_2_ beat^−1^)	25 ± 1	25 ± 0	26 ± 1	27 ± 2	28 ± 1	27 ± 1	27 ± 1
RER	1.15 ± 0.06	1.16 ± 0.06	1.13 ± 0.01	1.15 ± 0.02	1.17 ± 0.05	1.12 ± 0.02	1.16 ± 0.01
HR (beats min^−1^)	184 ± 4	179 ± 9	181 ± 2	180 ± 2	186 ± 3	178 ± 6	183 ± 1
PO (W)	352 ± 10	411 ± 51	388 ± 26	397 ± 34	426 ± 34	395 ± 40	424 ± 32
PO (W kg^−1^)	4.4 ± 0.1	5.0 ± 0.7	4.7 ± 0.3	4.8 ± 0.4	5.2 ± 0.4	4.8 ± 0.5	5.2 ± 0.4

*Note*: Number of participants *n* = 3. Abbreviations: *f*_R_, respiratory frequency; PO, power output; RER, respiratory exchange ratio; ${\dot{V}}_{E}$, pulmonary ventilation; ${\dot{V}}_{O_{2}}$, oxygen uptake; *V*_T_, tidal volume.

## 4 DISCUSSION

This paper reports temporal changes in the cardiorespiratory fitness profile of three world‐class ILCA‐7 sailors in the period of 2015–2020. In the realm of competitive sport, data collected during this period suggest moderate‐to‐high levels of cardiorespiratory fitness. Concurrently, spanning the period from 2015 to 2020, these three athletes secured a total of 21 medals at major international competitions. Their ${\dot{V}}_{O_{2}\mathrm{peak}}$ ranged from 51.7 to 61.9 mL min^−1^ kg^−1^ (refer to Table 2 and Figure 2 for individual readings) and fluctuated by approximately 10 mL min^−1^ kg^−1^, synchronizing with the international competition schedule, and the training plan with periods of moderate and intense workouts, with variations in both load and duration. During transitional phases, the training load typically ranged from 40 h per month, while in preparatory periods, it increased to 50–55 h. The peak value of ∼62 mL min^−1^ kg^−1^ was recorded in November 2019, when these athletes were nearly ∼30 years old, arguably reaching their potential. Other studies have indicated that world‐class Olympic athletes reach their peak aerobic performance between the age of 20 and 25 years, and can maintain high levels of oxygen uptake despite ageing effects on their cardiovascular system (Mikulic & Bralic, 2018; Nybo et al., 2014). Similar ${\dot{V}}_{O_{2}\mathrm{peak}}$ readings were reported for Danish sailors (Bojsen‐Møller et al., 2015, 2007). Specifically, the Danish authors provided data covering seven classes of sailing boats. Categorized into four distinct male groups, ILCA‐7 sailors demonstrated the highest ${\dot{V}}_{O_{2}}$ at 58.3 ± 4.2 mL min^−1^ kg^−1^, while the Finn and Star sailors exhibited the lowest at 47.6 ± 3.5 mL min^−1^ kg^−1^. However, differences in relative and absolute ${\dot{V}}_{O_{2}}$ among various sailing classes should be considered in the context of stature and body composition. For example, Finn sailors are typically ∼20 kg heavier than ILCA‐7 sailors, which means that heavier sailors have higher absolute ${\dot{V}}_{O_{2}}$ and rely more on absolute power (and muscle mass contribution) for pumping, a crucial factor in performing well downwind.

A retrospective analysis conducted by the same group of authors, involving approximately 400 individual CPET on around 120 sailing athletes over a 25‐year period, demonstrated that peak ${\dot{V}}_{O_{2}}$ in ILCA‐7 sailors ranged from 57 to 64 mL min^−1^ kg^−1^, highlighting the importance of well‐developed aerobic power within this group (Bojsen‐Møller et al., 2015). This could be ascribed to the diverse aspects of competitive LCA‐7 sailing, where competitive success is dependent on understanding weather conditions, the calibre of equipment, technical proficiency and strategic prowess (Winchcombe et al., 2021), underlined by high levels of aerobic power enabling these athletes to excel and win medals at international tournaments.

One of the most intriguing observations of this work was the alterations in a surrogate marker of stroke volume, namely oxygen pulse, observed over the course of the study. In 2015, we recorded an oxygen pulse of 25 mL O_2_ beat^−1^, at the age of 26 ± 3, and over the subsequent 6 years, the O_2_ pulse marginally increased and appeared to stabilise at 27 ± 1 mL O_2_ beat^−1^ in 2020, when these athletes were 32 ± 3 years old (Figure 2). This outcome was rather unexpected, as a linear increase over time was expected based on previous research on cardiovascular adaptations in elite athletes. For instance, a study by Mikulic and Bralic (2018) reported an increase of 1 mL O_2_ beat^−1^ year^−1^ over an 11‐year longitudinal period in elite rowers, while Nybo et al. (2014) documented a gradual rise in oxygen pulse in elite rower throughout his competitive career (with the final data collection occurring when the athlete was 40 years old). One possible explanation for the observed difference lies in the distinct nature of Olympic rowing and ILCA‐7 sailing. The ILCA‐7 sailing involves a significant proportion of isometric and quasi‐isometric contractions, likely contributing to substantial blood vessel occlusion due to intramuscular mechanical and intrathoracic pressure development. This, in turn, leads to compensatory adjustments in stroke volume over time (Mueller et al., 2018). According to the study of Lopez et al. (2016), which evaluated the cardiovascular parameters of Optimist class sailors, MAP and total peripheral resistance were elevated by 39% and 50%, respectively, during on‐water sailing compared to laboratory measurements obtained through CPET. However, our hypotheses need additional confirmation since no ultrasound data of the heart were collected to verify this assertion. Further research in this direction is, therefore, warranted, especially since the effects of ageing were not extensively evaluated in the context of Olympic sailing.

One novelty of this work, compared to previous research on the cardiorespiratory fitness profile of ILCA‐7 sailors, is the analysis of ventilatory thresholds. We observed ${\dot{V}}_{O_{2}}$ ranging from 40 up to 47 mL kg min^−1^ at GET, and 47 up to 54 mL min^−1^ kg^−1^ at RCP. In general, variations in physiological responses at ventilatory thresholds seem to be proportional to the alterations in peak oxygen uptake. This serves as a gross indicator of adjustments in buffering capacity and muscle recruitment patterns, among other adaptive mechanisms. As presented in this work, these sailors were apparently able to reach the GET and RCP at 70%–75% and 85%–90% of their maximal ${\dot{V}}_{O_{2}}$ (refer to Table 1 for mean values, and Figure 1 for individual readings), resembling data observed in elite endurance athletes (Laursen et al., 2003; Pallarés et al., 2016; Rodríguez‐Marroyo et al., 2017). Since the analysis of ventilatory thresholds has been shown to accurately track improvements in endurance performance in elite athletes, and in parallel obtaining valid and consistent on‐water data recording is rather limited, Cunningham and Hale (2007) conducted a 30‐min simulation protocol in a dinghy ergometer. They observed mean oxygen uptakes of nearly 60% of ${\dot{V}}_{O_{2}\mathrm{peak}}$ (∼2.5 L min^−1^), with heart rates of 155–160 bpm corresponding to ∼80%–85% of HR_max_, in six experienced sailors. Similar HR readings at GET were observed here (Table 1 and Figure 1), thereby indirectly validating the application of laboratory testing in exercise prescription for elite sailors. These data are also aligned with actual on‐water HR of up to 150–168 bpm^−1^ collected during upwind hiking in the ILCA‐7 (Vogiatzis et al., 1995), supporting the concept that hiking in the ILCA‐7 requires a well‐developed maximal aerobic power, apparently to a greater extent than common wisdom might suggest. Nevertheless, exercise prescription based on this traditional approach (% change in exercise intensity based on ${\dot{V}}_{O_{2}}$ or HR_max_) is often criticized due to the inherent limitations of this method (Gifford & Larsen, 2023). Primarily, this is due to the heterogeneity in individual responses to the fixed percentage of exercise load and the fact that changes in ${\dot{V}}_{O_{2}\mathrm{peak}}$ are not necessarily consistent with changes in physiological thresholds. Therefore, to provide deeper insight and optimize an individual performance of world‐class laser‐class sailors, a more individualized approach is warranted, with the determination of ventilatory thresholds for each athlete to improve maximal aerobic power, while avoiding premature fatigue development and suboptimal performance.

### 4.1 Study limitations

The onset of the Covid‐19 pandemic and subsequent lockdowns disrupted certain data collection plans, including those scheduled just before the Tokyo 2020 (held 2021 actually) Olympics, potentially affecting the generalizability of our conclusions. Further research is necessary to grasp the physiological foundations and individual variations contributing to changes in oxygen pulse concerning ageing effects in world‐class sailors. The implementation of more comprehensive data collection protocols over longer time periods is certainly warranted. From the perspective of clinical medicine, an ultrasound of the heart should be employed to provide better insight into cardiovascular adaptation. From an exercise physiology standpoint, an assessment of endurance at different intensity domains, including critical power, will be necessary to optimize strength and conditioning programs and enhance sport performance.

### 4.2 Conclusion

This paper investigates the temporal changes in the physiological profiles of three ILCA‐7 sailors, all of whom have previously won medals at major international competitions. The present findings suggest that a well‐balanced aerobic power is essential for superior performance in ILCA‐7 class sailing. This work stands out as it offers a deeper insight into the cardiorespiratory fitness of world‐class Olympic sailors by analysing ventilatory thresholds, delving beyond a mere assessment of ${\dot{V}}_{O_{2}\mathrm{peak}}$ alone. Oxygen pulse readings were analysed over a 6‐year period, revealing a lack of notable changes throughout the study. This suggests the involvement of compensatory cardiovascular mechanisms, likely associated with the isometric and quasi‐isometric contractions inherent in ILCA‐7 sailing. Further research should investigate whether this serves as a marker of compensatory mechanisms specific to this athlete population and its relationship to ${\dot{V}}_{O_{2}\mathrm{peak}}$, utilising more comprehensive research tools.

## AUTHOR CONTRIBUTIONS

All authors conceived this study. Vladimir Ivančev conducted the experiments, and both Vladimir Ivančev and Damir Zubac analysed the data. The manuscript was written by all authors. All authors have read and approved the final version of this manuscript and agree to be accountable for all aspects of the work in ensuring that questions related to the accuracy or integrity of any part of the work are appropriately investigated and resolved. All persons designated as authors qualify for authorship, and all those who qualify for authorship are listed.

## CONFLICT OF INTEREST

None.

## FUNDING INFORMATION

None.

## Data Availability

The data that support the results of the present work are available from the corresponding author upon reasonable request.
